# Do the Walkability and Urban Leisure Amenities of Neighborhoods Affect the Body Mass Index of Individuals? Based on a Case Study in Seoul, South Korea

**DOI:** 10.3390/ijerph17062060

**Published:** 2020-03-20

**Authors:** Yunwon Choi, Heeyeun Yoon

**Affiliations:** 1 Interdisciplinary Program in Landscape Architecture, Seoul National University, 1 Gwanak-ro, Gwanak-gu, Seoul 151-742, Korea; yunwon.choi@snu.ac.kr; 2 Department of Landscape Architecture and Rural Systems Engineering, College of Agriculture and Life Sciences, Seoul National University, 1 Gwanak-ro, Gwanak-gu, Seoul 151-921, Korea; 3 Research Institute of Agriculture and Life Sciences, Seoul National University, 1 Gwanak-ro, Gwanak-gu, Seoul 151-921, Korea

**Keywords:** obesity, walkability, urban leisure amenities, structural equation modeling

## Abstract

This study investigates the impact of neighborhood-built environments on obesity in interrelationship with socioeconomic status (SES)—controlling for dietary patterns and physical activities of residents—using structural equation modeling (SEM). A total of 577 samples who are between 19 and 64 years old and reside in Seoul are extracted from Korea National Health and Nutrition Examination Survey (KNHNES), 2015. Neighborhood environments are represented as the two latent constructs—walkability and leisure amenities—composited with indicators such as density of intersections, density of mixed-use area, and the area of open spaces and are aggregated by jurisdictional unit in Seoul. We found that greater walkability in a neighborhood explained a lower body mass index (BMI) among residents, whereas more urban leisure amenities in a neighborhood explained a higher BMI. The finding suggests that a walking-friendly environment is more effective than active recreational amenities in inducing people to engage in daily physical activities to the level that reduces obesity rate. SES exerted a negative impact on BMI of a greater magnitude than the impact of either of the environmental living conditions, reinforcing the importance of general wealth and education level in leading to a healthy lifestyle. Our research contributes to growing evidence of a relationship between obesity and the built environment in the context of Asian countries where the prevalence of obesity is becoming a serious issue and requires immediate attention.

## 1. Introduction

Obesity is a condition of excessive fat accumulation that poses higher risks of developing serious medical illnesses such as hypertension, high cholesterol, diabetes, arthritis, cardiovascular diseases, and some forms of cancer [1]. Obesity has become one of the major challenges in human health globally [2], not only because it has been increasing at an alarming rate but also because reportedly no country has been able to decrease the obese population successfully, over more than three decades, as of 2013 [3]. According to WHO’s 2014 data, about 39% of the adult population worldwide suffers from overweight problems, and at least 3.5 million of the annual death is attributed to the relevant reasons [2]. A rapid increase in prevalence of obesity was discovered in Asian countries and the changes in lifestyle and eating habits were pointed out as main causes [2]. South Korea is no exception. Currently, the rate of obesity is not as high there as in other developed countries, but it is steadily increasing. As of 2016, one in three adults was overweight, and this number is expected to grow even more severely in the future, because Koreans have adopted a Western-style diet [4].

With the rising prevalence of obesity, experts—mostly in the fields of medicine and human biology—made efforts to identify the causes; obesity has been attributed to genetic propensities, diseases, drugs, and lifestyles [5]. Recent studies have discussed environmental conditions as an important factor, because they affect the lifestyles of individuals [6]. Some new initiatives, such as Healthy City of Healthy People, have formed to help reduce obesity rates by improving built environments [7]. These initiatives are international or national programs promoting local strategies for health protection and sustainable development and elimination of health disparities [8,9]. To achieve these goals, researchers and planning practitioners have found it effective to supply more pedestrian-friendly and leisure-inducing amenities throughout cities.

In a walkable community, hypothetically, more people would tend to walk, and consequently fewer people would be obese [10]. A pedestrian-friendly environment commonly includes well-connected streets with frequent intersections and adequately sized blocks [11,12], mixed land use with a wide variety of destinations [13], and easy access to public transportation [14]. Researchers also found that a higher level of urban leisure amenities is associated with a lower level of obesity incidence [15]. In particular, numerous nearby parks are known to encourage physical activity, decreasing obesity incidence [16].

The occurrence of obesity also depends on the social context [17]. In Western countries, a large portion of the obese population is found in low-income classes. People with higher socioeconomic status (SES) tend to feel relatively more pressure to be fit and to pursue a healthy lifestyle than people with lower SES, so they make more effort to have an appropriate diet and physical activities. This difference is magnified by their differing living environments [18,19]. First, low-income communities have relatively few public amenities—such as playgrounds, sidewalks, and recreational facilities—that potentially encourage physical activities, in comparison to places where tax money is abundant [20,21,22,23]. Second, low-income neighborhoods have lower accessibility to healthy food resources and higher accessibility to fast food, to which the residents routinely resort, resulting in poor dietary habits [24,25].

While studies have attributed the incidence of obesity to these individual and discrete factors, none has examined in combination the direct and indirect interrelationships among obesity, neighborhood environment, SES, and lifestyle [10,26,27,28]. To understand the impact of neighborhood environments on the obesity level of the residents, other conditions need to be considered simultaneously, as they might have interconnected relationships with the neighborhood environments. In particular, as mentioned above, SES is strongly related to lifestyle and living environment, some elements of which would influence the level of obesity directly while others would do so only in an indirect way. This circumstance might differ by geographic region, but a majority of studies on this subject have been conducted in Western countries. The findings of the previous literature are thus difficult to apply to Eastern countries where the prevalence of obesity is becoming a serious issue and requires immediate attention [29].

Against this backdrop, we investigate the impact of neighborhood-built environments on obesity in interrelated relationships with SES, controlling for dietary patterns and physical activities of residents, using structural equation modeling (SEM). The study site is Seoul, South Korea, and the study period is January 1, 2015 to December 31, 2015.

The structure of this text is as follows. In the next section, we briefly review the literature on the built and social environment as components of a healthy city. After explaining our analytical design, including research question, study site, data, variables, and methodology, we then describe the results. The final section discusses the implications of our findings, limitations, and future research direction.

## 2. Materials and Methods

### 2.1. Research Question

Our research question is: Does the neighborhood environment, along with socioeconomic characteristics, impact individuals’ degree of obesity, controlling for dietary pattern and physical activity? BMI is our main dependent variable; neighborhood environments, represented as walkability and leisure amenities, are the question variables. We use SEM (structural equation modeling) to decipher this interrelationship among constructs: walkability (walkability), urban leisure amenities (urban leisure amenities), SES (SES), dietary pattern (dietary pattern), and physical activity (physical activity).

The two hypotheses in this research are:

**Hypothesis** **1.** *Greater walkability in a neighborhood explains lower BMI of the residents*.

**Hypothesis** **2.** *More urban leisure amenities in a neighborhood explain lower BMI of the residents*.

### 2.2. Study Site

**Figure 1:**
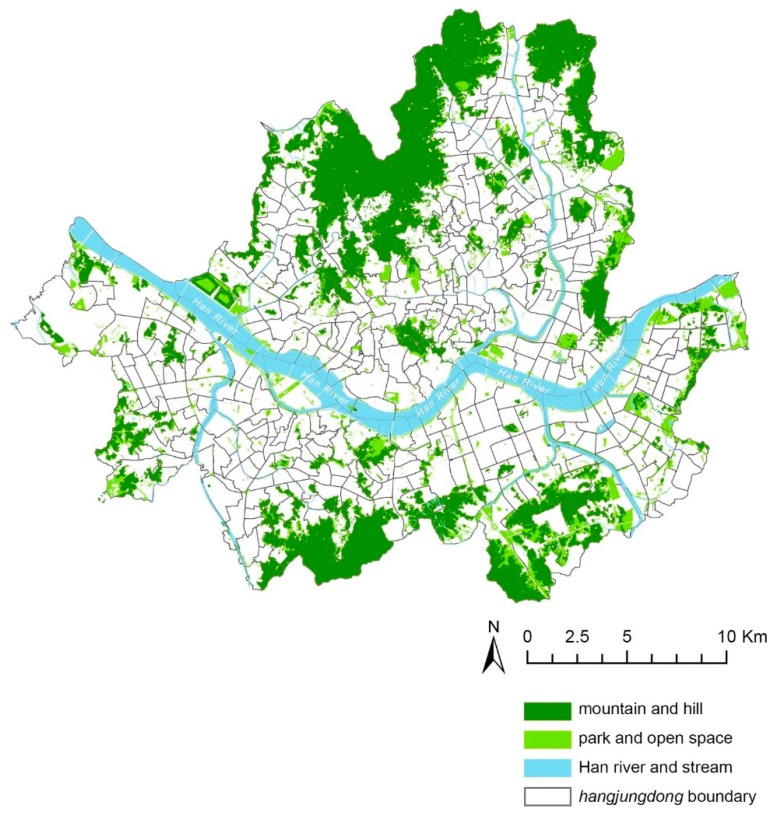
The study site: Seoul, South Korea.

The study site is Seoul, South Korea (Figure 1). Seoul accounts for only 0.6% of the total area of South Korea, but it is densely populated, with almost one-fifth of the national population (about 10.2 million live in the city). Seoul contains 486 hangjungdong (HJD), units of administrative districts, of which the average area is 1.33 km^2^ (min = 0.42 km^2^, max = 2.93 km^2^). Geographically, Seoul is a nature-rich city with mountains, rivers, and streams. Large mountains surround the periphery of the city, whereas small hills are situated throughout the city [30,31]. A multi-modal public transportation system is well established. Buses and subways operate in most areas of the city, making it easy to commute [31,32].

Although Seoul is mostly built up to its capacity, it supplies abundant urban leisure amenities and the opportunities for physical activities to its citizens, taking advantage of its rich natural resources. In the mountains and hills, well-paved hiking trails are available. Moreover, by the Land Development and Utilization Act, most of the sizable housing complexes of the city contain community spaces within or near their locations [33,34]. In addition, most sections of the rivers and streams are flanked with riverfront parks and esplanades that citizens utilize to walk, bike, and picnic.

The Han River divides the city into two parts, both geographically and socially: older towns are located north of the river, and the newer and wealthier neighborhoods are located south of the river [32,35]. Because the northern part of Seoul has been developed as an administrative and economic core of the city for about six hundred years, it retains a historic urban form represented as small-grained and organic street networks and block systems [36]. On the other hand, the southern part of the city has been developed later than the northern part, planned by national and city governments since 1970s [37]. A large part of the area adopts the superblock system, and the roads and streets are more regularly laid out (Figure 1).

### 2.3. Data and Samples

Three primary datasets were used in this study: Korea National Health and Nutrition Examination Survey (KNHNES) 2015, Statistical Geographic Information Service (SGIS) provided by Statistics Korea, and digital maps provided by the Korea National Geographic Information Institute.

#### 2.3.1. Korea National Health & Nutrition Examination Survey (KNHNES)

KNHNES is a national health and nutrition survey that is conducted to understand public health and nutritional status under Article 16 of the National Health Promotion Act. It includes health examination records, health interviews, nutrition records and personal information related to socioeconomic status. The health examination records include individuals’ information such as obesity, high blood pressure, diabetes, dyslipidemia, liver disease, kidney disease, anemia, lung disease, oral disease, muscle strength, eye disease and otolaryngology disease. The health interview part includes household surveys, education, economic activity, smoking, drinking, obesity and weight control, physical activity, morbidity, medical use, vaccinations and health screenings, limited activities and quality of life, impairment (accident and addiction), safety awareness, mental health, women’s health and oral health. Nutrition record includes food and nutrient intakes, dietary behavior, dietary supplements, nutritional knowledge, food stability, feeding status, weaning supplements. The survey subjects are about 10,000 people from 3840 households randomly selected from the 192 sampled survey sites in South Korea every year. The nutrition records indicate the estimated calorie and nutrient intakes of respondents from the 120 types of foods that are highly consumed in Korea. We used KNHNES, collected in 2015, to gain information on individual characteristics such as demographic information, BMI, SES, dietary pattern, physical activity, and addresses. Out of 7380 individuals, we selected 577 who are between 19 and 64 years old and reside in Seoul [38].

#### 2.3.2. Built Environmental Data

We collected built environment data from various sources. A digital map was sourced from the National Geographic Information Institute to identify the locations of bus stops, intersections, residential complexes, and bike lanes. For subway stations and school playgrounds, we referred to data from the Statistical Geographic Information Service (SGIS). From the biotope map provided by the Seoul Metropolitan Government, we extracted information about land use, community sports facilities, open spaces, and parks. Information on hiking trails was provided by the Korea Forest Service. Population density was collected and provided by Seoul Statistics. The analytical unit is a person (an individual). Environmental characteristics are aggregated by HJD, due to information privacy laws that the institutes must abide by.

### 2.4. Statistical Analysis: SEM

We employed SEM to answer the research questions via statistical analysis software, AMOS 22 (IBM, Chicago, IL, USA). SEM functions as a combination of multiple statistical techniques—factor analysis, multiple regression, and path analysis—in one model and is used to simultaneously assess intertwined relationships among constructs. The several significant benefits of the technique have led its application to many different fields of study. The first strength of SEM is its capacity to deduce complex relationships simultaneously among multiple endogenous and exogenous latent constructs, while each latent construct is measured with multiple indicators [39]. In addition, unlike multiple regression models, SEM differentiates between those relationships as causal or association, direct or indirect. The second strength of SEM is its unbiased estimation of parameters. Unlike a traditional regression model, SEM considers measurement error, allowing the presence and correlation of error for each observed indicator in the model. In addition, as with a traditional regression model, the unexplained part of variance of a dependent variable, here called structural error in SEM, is specified in the model. Therefore, with these errors controlled, the parameter estimates are unbiased [40].

**Figure 2:**
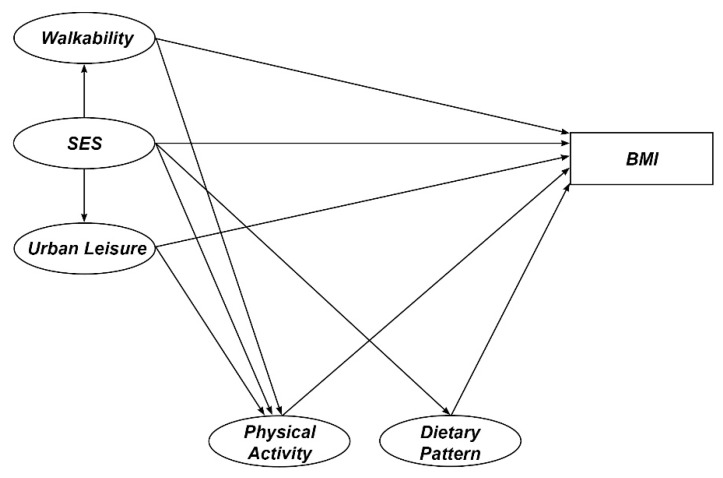
Research model.

We specified an SEM model to test whether the BMI—the endogenous indicator—can be explained by five constructs. We hypothesized that walkability and urban leisure provision would affect individuals’ BMI, and we attempted to identify the role of SES among those three constructs. Dietary pattern and physical activity were controlled so we could evaluate the unconvoluted impact of those two neighborhood environmental constructs on BMI. The research model is presented in Figure 2.

### 2.5. Statistical Analysis: Constructs and Indicators

Our dependent indicator is BMI. BMI is a universal measure of an individual’s body mass, divided into four categories: underweight, normal weight, overweight, or obese. The value is calculated as follows:

(1) $$BMI=\left(weight\;in\;kilograms\right)/{\left(height\;in\;meters\right)}^{2}$$

WHO regards a BMI less than 18.5 as underweight, BMI equal to or greater than 25 as overweight, and BMI above 30 as obese (WHO Expert Consultation, 2004). However, because genetic differences can affect body fat patterns, different criteria are adopted for different ethnic groups. In Asia, BMI less than 18.5 is considered underweight and BMI of 18.5–23 represents an increasing but acceptable risk of being obese, 23–27.5 a heightened risk of being obese, and 27.5 or higher a high risk of being obese [41].

#### 2.5.1. Exogenous Constructs: Neighborhood Built Environment and SES

##### Walkability

Walking is one of the most effective forms of moderate to vigorous physical activity to promote health for all age and gender groups, because it is relatively safe, easy, and free [42]. Previous studies revealed that people who lived in a more walkable neighborhood walked more frequently and consequently had lower body mass than those living elsewhere [43,44]. In Baltimore, Maryland, people living in high-walkability neighborhoods had 92% longer walking time, 97% longer bicycling time, and 13% shorter vehicle time compared to others who live in low-walkability neighborhoods [43]. In Belgium, living in a high-walkability neighborhood induced 74% more walking, specifically 33.4% more recreational walking, 44.7% more cycling to destinations, and 3% more physical activities [45]. A study analyzing anthropometric data from UK Biobank suggested a negative association between active commuting and the obesity level of people of middle age. It is confirmed that people commuting by public transportation, by bike, or on foot had significantly lower percentage of body fat—approximately from −3.26% to −1.10%—compared to those driving to work [46]. Walkability is a measure of the pedestrian friendliness of the subject environment [47]. Although there is not a single universal set of indexing for walkability, the concept commonly includes three main components: residential density, land-use mix, and street connectivity [48]. According to a previous study investigating 10,008 participants in 14 cities of 10 countries from the International Physical Activity and Environment Network (IPEN) adult study, people living in neighborhoods with higher residential density, more intersections, and more public transportation tend to engage in 0.6%, 6.9%, and 3.7% more physical activities, respectively, than those who live in other neighborhoods [49].

**Figure 3:**
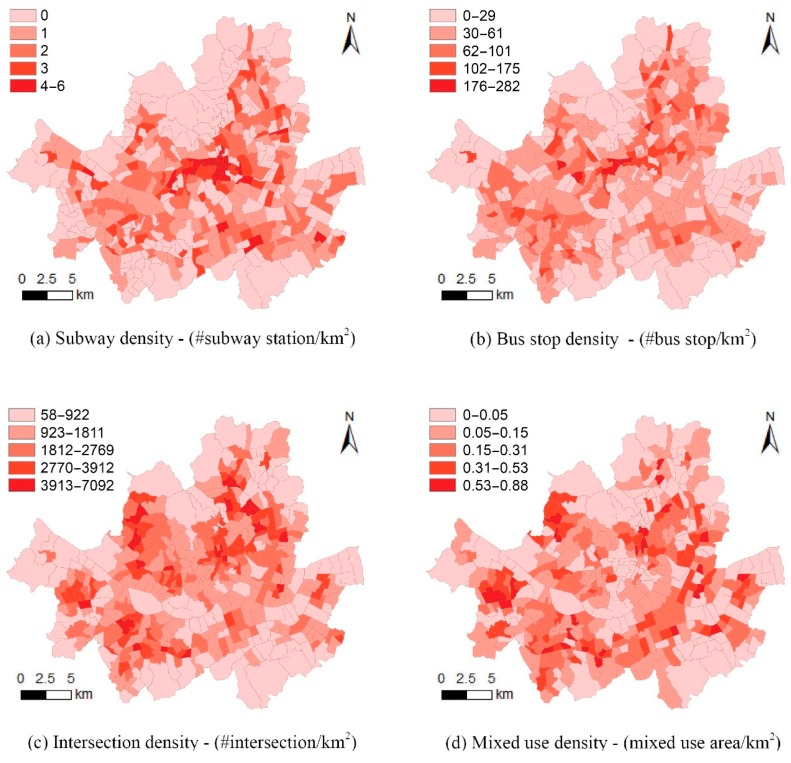
The present condition of the walkability components.

The construct Walkability indicates the pedestrian friendliness of the road and street system of each HJD. To construct Walkability for each HJD, we used five measured variables including residential density (residential density), subway station density (subway density), bus stop density (bus stop density), proportion of mixed land use (mixed land use), and intersection density (intersection density). The variable residential density is the number of residents per unit area of an HJD (km^2^), which indicates how densely populated the HJD is. The variables subway density and bus stop density measure the numbers of those stations and stops per unit area of an HJD (km^2^). The variables mixed land use and intersection density estimate the ratio of the mixed-use area to the total area and the number of intersections per unit area of an HJD (km^2^), respectively. The present condition of the walkability components of each HJD of Seoul is presented in Figure 3.

##### Urban Leisure Amenities

Besides walkability, urban leisure amenities represent another important element of healthier built environments [50,51,52,53,54]. Leisure amenities, such as parks, bike paths, trails, sports facilities, playgrounds, and open spaces, provide residents opportunities for various physical activities. Several studies have revealed that urban leisure amenities in a residential area are closely related to the physical activity level of residents, who hence have lower body mass index (BMI). For Australian and U.S. women, having a higher number of parks near home was associated with 4% and 35% lower odds, respectively, of being overweight/obese [55]. This relationship was also found among children. In the United States, children living in neighborhoods with access to parks or facilities have 21% lower prevalence of obesity than children in otherwise-neighborhoods [56].

In addition to the accessibility and proximity of parks, the types and quality of park features have also been highlighted in many studies [57,58,59,60,61,62,63,64]. In medium-sized Canadian cities, children who have playground-equipped parks within 1 km of their homes were five times more likely to have a healthy weight than those in opposite conditions [65]. Furthermore, the presence of poorly managed parks in zip-code-level communities in New York City was associated with 18% higher BMI among the residents than those in the neighborhoods with well-managed parks. Therefore, efforts should be made to create safe and clean environments to encourage physical activities [44].

The construct—urban leisure amenities—measures the level of leisure amenities provided in each HJD. We composited the construct using eight measurement indicators, including a proportion of apartment complexes in residential areas (apartment complex), the total length of bike lanes in an HJD divided by the area of the HJD (bike lane density), the ratio of the total area of large parks (larger than 1 ha) and small parks (smaller than 1 ha) to the area of an HJD (large park density, small park density, respectively), the average number of community sports facilities per km^2^ of an HJD (sport facility density), the ratio of the total area of school playgrounds to the area of an HJD (school playground density), the area of open space—including all public parks—per person (open space), and the total length of hiking trails in an HJD divided by the area of the HJD (trail density).

##### SES

According to recent studies, SES is strongly associated with obesity and being overweight, but the nature of the relationship varied due to the differing definitions of economic levels in different countries [2]. In developed countries, people with lower income level are more likely to become obese [66,67]; they tend to eat low-cost fast foods that are unhealthy and fat-rich more frequently than healthy foods that are usually more expensive, whereas people with higher SES tend to be more conscious of their health and make efforts to eat healthily and exercise regularly [24,68]. Consequently, better food resources and restaurants with healthful menus are more available in wealthier communities [69]. In New York City, for those earning higher incomes, living with greater subway accessibility was consistently associated with a 35% lower BMI [70].

The construct SES indicates the economic and social position of an individual. We used two indicators to construct SES; the total years of education completed (education) and the average monthly household income of each sample recorded in approximated Korean won (household income).

#### 2.5.2. Control Constructs: Individual Characteristics

##### Dietary Pattern

Food culture changed during the 20th century, and people frequently eat out and consume more packaged food and fast food than before. Professionals argue that consuming processed foods might be unhealthy because the food industry commonly puts economic profits before the quality of ingredients and balance of nutrition [71]. Studies have revealed that manufactured food had poorer nutritional quality and more calories, which can result in increasing body mass and higher level of health problems [72]. Brazilian adults and adolescents who consume the highest quintile of ultra-processed foods had 94% higher body mass, 198% higher odds of being obese, and 126% excess weight, compared with those in the lowest quintile of such consumption [73].

The construct Dietary Pattern measures individuals’ dietary habits and is constructed with the following indicators: the number of times individuals eat out per week (eat out) and the total daily intakes of carbohydrate (carbohydrate), protein (protein), fat (fat), and sodium (sodium).

##### Physical Activity

The construct—physical activity—measures the types and the frequency of physical activities in which each individual takes part. It comprises the following eleven indicators: the total days of a weekly routine that included vigorous occupational physical activities (weekly vigorous work), moderate occupational physical activities (weekly moderate work), vigorous recreational physical activities (weekly vigorous recreation), moderate recreational physical activities (weekly moderate recreation), active commuting (weekly active commuting), and walking exercise (weekly walking) and the total hours of daily routine of vigorous occupational physical activities (daily vigorous work), moderate occupational physical activities (daily moderate work), vigorous recreational physical activities (daily vigorous recreation), moderate recreational physical activities (daily moderate recreation), and active commuting (daily active commuting).

Appendix A describes all latent variable formed with multiple measured variable used for the analysis (Table A1).

## 3. Results

### 3.1. Characteristics of Samples

Of the 577 observations in the samples, 38.1% are for male subjects and 61.9% for female subjects; the age range is 19–64, and the age distribution is 19–29 (19.2%), 30–39 (19.4%), 40–49 (25.7%), 50–59 (24.3%), 60–64 (11.4%); 5.7% of subjects are underweight (BMI below 18.5), 41.4% are normal weight (BMI between 18.5 and 23), 21.2% are overweight (BMI between 23 and 25), and 31.7% are obese (BMI above 25).

### 3.2. Estimating Measurement Models

When compositing the five latent constructs—walkability, urban leisure amenities, SES, dietary pattern, and physical activity—indicators with either multicollinearity, low internal consistency reliability or lack of statistical significance were excluded. For the construct walkability, we kept the three indicators bus stop density, intersection density, and mixed land use, and we eliminated the rest based on the result of construct reliability testing. For the construct urban leisure amenities, only the open space and trail indicators were adopted for the same reason. Table 1 describes the factor loading of indicators, Cronbach’s alpha value, and AVE (Average Variance Extracted) values of each construct. All constructs maintain acceptable Cronbach’s alpha and AVE values. For dietary pattern and physical activity, unlike our question latent variables, we retained indicators that did not explain the latent variable at a 5% level of statistical significance to control the impact of individual characteristics on BMI.

Finally, the model fit of our structure with the five individual constructs was evaluated based on the fit indices: χ^2^(CMIN/df) = 1.880, GFI (goodness of fit) = 0.946, CFI (Comparative Fit Index) = 0.967, RMSEA (root mean square error of approximation) = 0.039, TLI (Tucker–Lewis Index) = 0.958.

We have set the covariances between measurement error terms of indicators that follow the uppermost limit of modification index at 4.00, all of which are statistically significant at a 5% level. Since data used for SES, dietary pattern, and physical activity came from the same source, KNHNES 2015, correlation can occur between measurement errors of indicators from those three different constructs.

### 3.3. Analytical Results

**Figure 4:**
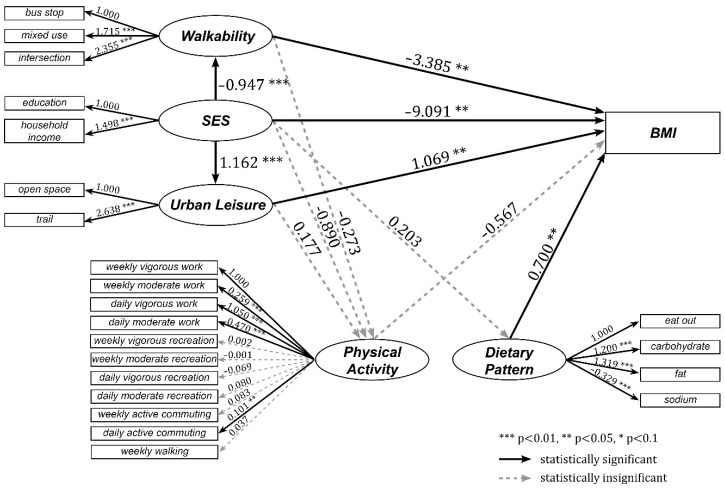
The results of analysis of how neighborhood environment effects BMI.

We present the results of SEM analysis in Figure 4. From our two question latent constructs, walkability of the neighborhood-built environment has influences on lower BMI of individuals, while urban leisure amenities has influences on higher BMI at a statistical significance level of 5%.

Walkability was negatively associated with BMI (coeff. = −3.385), meaning that if the walkability of the one’s neighborhood is higher, one’s BMI tends to be lower. This result agrees with the conventional wisdom that people living in a more walkable community have lower BMI [74]. Among the three indicators of walkability, the density of the intersections has the highest explanatory power in leveraging up the walkability (coeff. = 2.355), perhaps because it can provide various shortcuts to destinations and enrich walking experiences [75]. The variable mixed land use also has a large magnitude explaining the lower BMI (coeff. = 1.715), because such conditions put multiple destinations in close proximity [76], so that pedestrians tend to walk more, and the obesity rate may decrease.

The influence of urban leisure amenities over BMI seems to contradict the general expectation that providing urban leisure amenities will increase people’s opportunities for physical activity and help them lose weight [15]. Urban leisure amenities in the current model is positively explained by the provision of open space (coeff. = 2.638). We cannot rule out the possibility that in Seoul the conventional wisdom fails to apply because a mere presence of open spaces, such as large or small parks and children’s parks, does not induce enough physical activity to be effective in reducing weight. The latent urban leisure amenities and physical activity do not have a statistically significant relationship, supporting that claim. This observation might imply a limitation of the variable specification. The provision of open space does not mean people actually use such amenities [22]. The simple presence of amenities may not lead residents to “visit and use” [77]. In particular, some open spaces are still far from some parts of the HJD and/or unattractive to visit due to a poor level of management or lack of enough equipment, thus the use of the open space by residents would not be guaranteed [58,62,78]. Therefore, to clarify the relationship between urban leisure amenities and BMI, more evidence on actual use is needed.

Finally, SES has a negative effect on BMI (coeff. = −9.091), from which we can interpret that the economically well-off are relatively less obese. The magnitude of the impact is greater than that of walkability and urban leisure amenities on BMI. Among the indicators we initially specified, household income positively explains SES (coeff. = 1.498). This result corresponds to that of previous studies, in that people with higher SES have a lower obesity rate [18]. SES showed a statistically significant relationship with walkability and urban leisure amenities, which also aligns with previous findings, maintaining the presence of spatial disparity in the provision of a healthier living environment [21,22,23,79,80]. The SES is negatively associated with walkability (coeff.= −0.947), meaning that, on average the people of higher SES live in less walkable neighborhoods. Thus, it is not necessarily true that people with higher SES live in an environment that would induce everyday physical activities. On the other hand, the SES is positively associated with urban leisure amenities (coeff. = 1.162), revealing that more urban leisure amenities are supplied in wealthier neighborhoods than elsewhere. As mentioned above, it is in question whether such a quantity of urban leisure amenities would help reduce weight in the wealthier communities. Previous studies have disproved the claim and suggested that the provision of green spaces may yield more health benefits for low-SES groups and ethnic minorities [81,82,83].

To decipher the relationship between the neighborhood-built environment (walkability and urban leisure amenities) and SES, a specific settlement pattern in the city of Seoul should be acknowledged. As mentioned in the Background, the northern part of Seoul was developed earlier than the southern part and before today’s standards for urban planning had been officially adopted. The old neighborhoods in the northern part are composed of smaller, organically shaped blocks with higher population density, compared with new neighborhoods across the river [84]. These conditions may have made the neighborhoods more walkable. On the other hand, areas south of the river have been developed with modern urban planning since the late 20th century. Some neighborhoods developed under this approach contain rectangular superblocks centered on personal vehicles [85]. These conditions may have reduced walkability of the neighborhoods [86]. Currently, real-estate values are much higher in the southern part than in the northern part of the city, thus the south is inhabited by a wealthier group and the average SES is higher in the south than in the north. Because the south has more abundant tax money, more urban leisure amenities may have been provided in that region than in its northern counterpart.

## 4. Discussion

In this study, we aimed to reveal the impact of neighborhood-built environment on obesity with an interrelationship with SES, controlling for the individuals’ physical activity and dietary pattern, using SEM. In the analytical results, only one hypothesis (Hypothesis 1) was found to be true in the context of Seoul: greater walkability in a neighborhood explained lower BMI of the residents, whereas more urban leisure amenities in a neighborhood explained higher BMI. SES produced a much greater negative impact on BMI than environmental living conditions did. This result, in one of the largest cities in Asia, accords with the findings on the same topic from Western countries. Perhaps people with higher SES in Seoul may also (as in the West) consciously strive for a healthier lifestyle in terms of dietary pattern and exercise. However, that reasoning was not corroborated empirically in this study. Since the provision of urban leisure amenities and physical activities have no statistically significant relationship, we cannot conclude that supplying parks would encourage physical activities sufficiently to reduce the obesity rate.

Our result would be helpful in making the community environment for active living. In 2018, the WHO has reported that one in three South Korean adults lacks exercise [87]. Particularly 94% of Korean youth does so, which is the worst among the 146 countries [88]. Anecdotal evidences reported that young people are prone to engage in physically inactive activities such as web surfing, watching TV and mobile phones [89]. The WHO recommends adolescents one hour of exercise per day [90]. However, Korean elementary and junior high schools only have three hours of physical education a week, and high school is one to two hours a week. In particular, there are no physical education classes at all in the first and second year of elementary school, and many parents prefer their children to focus on academics rather than physical education. Experts pointed out that lack of adolescence can avoid exercise and increase obesity. Experts pointed out that neglecting exercise at school can undermine students’ awareness of exercise, and eventually Korean youth can avoid exercise and obesity rate can increase. To increase physical activities, it is important to encourage youth and also adults to exercise more either in their daily routine or during their recreation time [91]. Both utilitarian and leisure walking can easily become a daily routine for commuting, grocery shopping, and eating out. The effects of routine exercise would be larger than the effects of active recreational activities, such as going to parks or walking on trails, which are occasional at best and may not be sufficient to achieve health improvement. By providing convenient public transportation, increasing mixed land use, and improving street connectivity, municipalities can enhance the walkability of neighborhoods. Although this study did not reveal that people in walkable environments are indeed engaged in more walking, our analytical result of the relationship between lower BMI and higher neighborhood walkability implies that it is so. Additionally, encouragement and awareness talk of physical activities by peers and physical education teachers in school setting realities can be also helpful in increasing exercise using this environment [91,92].

When planning a park, it is important to provide easy access and enough attractive features, considering proximity to communities, generous size, quality play equipment, and the level of maintenance [93,94,95]. A larger number of urban leisure amenities alone does not necessarily result in a higher physical activity of residents [96], as our analysis shows. Depending on the conditions at the parks, people make decisions on whether to use the leisure amenities or not. Unlike street walking, visiting urban leisure amenities requires planning, time, and effort, and therefore amenities should be attractive enough to encourage usage [78].

Spatial socioeconomic equality should be also an important consideration in tackling the BMI problem. SES showed the greatest impact on BMI among latent variables. As mentioned earlier, people with differing SES have differing levels of concern about their health. Because the current analysis could not reveal the direct relationship among SES, dietary pattern, and BMI, identifying the mechanism of high SES influence upon lower BMI may be an important future research question.

## 5. Conclusions

In addition to analytical findings, our research contributes to growing evidence of relationships between the obesity problem and the built environment in the context of Asia and more specifically of South Korea. Due to the diversity of cultures and urban forms among Asian countries, researchers and institutions need to vigorously study the obesity problem and its factors in different Asian countries. Accumulated knowledge will be helpful in preventing rising obesity problems in Asia.

Experts can use our findings to promote public health for physical urban planning and policy-making. Pedestrian environments can be prioritized as an effort to create healthy and safe urban settings and encourage people to walk more in their daily lives. Some features to be considered are diagonal crossings at intersections, street trees for shade, wider sidewalks, and crime prevention through environmental design (CPTED) [97,98]. In addition, municipalities and practitioners should find a strategy to maximize the usage of urban amenities to the levels that help reduce the obesity rate. Joining urban amenities to walking routes, such as linear parks, would be one possible strategy in this case [99,100]. In addition, such amenities might be more effective in improving health conditions in low-income neighborhoods. Priority can be given accordingly in budget execution for public landscape projects.

This study can form the basis for many future studies. Substantial improvements are possible if more detailed data become available. For example, tracing residents’ movements and recording their dietary patterns and BMI would reveal a more solid relationship than this current study. In the nationwide survey data utilized in this study, information is only partially publicized due to the protection of personal information. Additionally, due to the aggregation of values by jurisdictional unit, a modifiable areal unit problem might have presented and affected the analytical results.

## Figures and Tables

**Table 1:** Measurement information and reliability checks.

Indicator	Factor Loading	Construct Reliability (α)	AVE (Average Variance Extracted)
**Walkability**		0.718	0.56
bus stop density	1.000 ^1^		
mixed land use	1.715 ***		
intersection density	2.355 ***		
**Urban Leisure Amenities**		0.726	0.72
trail density	1.000 ^1^		
open space	2.638 ***		
**Socioeconomic Status (SES)**		0.537	0.15
education	1.000 ^1^		
household income	1.498 ***		
**Dietary Pattern**		0.537	0.51
carbohydrate	1.000 ^1^		
fat	1.200 ***		
sodium	1.319 ***		
eat out	−0.329 ***		
**Physical Activity**		0.685	0.19
weekly vigorous work	1.000 ^1^		
weekly moderate work	0.259 ***		
daily vigorous work	1.050 ***		
daily moderate work	0.470 ***		
weekly vigorous recreation	0.002		
weekly moderate recreation	−0.001		
daily vigorous recreation	0.069		
daily moderate recreation	0.080		
weekly active commuting	0.083		
daily active commuting	0.101 **		
weekly walking	0.037		

^1^ Reference indicator, ** *p* < 0.05, *** *p* < 0.01.

## Appendix A

**Table A1:** Description of variables.

Latent Variable	Measured Variable	Description	Data Source	Sample (n)	*Mean*	*S.D.*	*Min*	*Max*
	BMI (Body Mass Index)	Dependent variable (BMI = weight (kg)/height(m)^2^)	KNHNES	577	23.55	3.67	15.80	37.60
**Walkability**	residential density	The number of residents per unit area of HJD (persons/km^2^)	SGIS		26,953.06	12,581.55	4941.00	62,200.00
	subway density	The number of subway stations per unit area of HJD (number of subway stations/km^2^)	SGIS		1.00	0.97	0.00	3.17
	bus stop density	The number of bus stops per unit area of HJD (number of bus stops/km^2^)	SGIS		8.73	5.43	0.00	27.78
	mixed land use	The ratio of mixed land use to area of HJD (area of mixed land use (km^2^)/total area of hangjungdong (km^2^))	SGIS		0.00	0.39	−0.34	1.25
	intersection density	The number of intersections per unit area of HJD (number of intersections/total area of hangjungdong (km^2^))	SGIS		842.02	530.49	45.87	2190.48
**Urban Leisure Amenities**	open space	The area of open space including all parks per person (total area of open space in hangjungdong (m^2^)/total number of residents in hangjungdong)	Digital map		1.58	1.72	0.00	8.10
	trail density	The length of hiking trails per unit area of HJD (total length of hiking trails in hangjungdong (m)/total area of hangjungdong (km^2^))	Digital map		1668.39	5294.45	0.00	30,436.77
	apartment complex	The proportion of apartment complexes in residential land use (total area of apartment complexes in hangjungdong (m^2^)/total area of residential land use of hangjungdong (km^2^))	SGIS		0.83	0.61	0.00	2.82
	bike lane density	The length of bike lanes per unit area of HJD (total length of bike lane in hangjungdong (m)/total area of hangjungdong (km^2^))	Digital map		1551.59	1793.66	0.00	7083.46
	large park density	The area of large parks per unit area of HJD (total area of large parks in hangjungdong (km^2^)/total area of hangjungdong (km^2^))	Digital map		0.03	0.06	0.00	0.38
	small park density	The area of small parks per unit area of HJD (total area of small parks in hangjungdong (km^2^)/total area of hangjungdong (km^2^))	Digital map		0.02	0.02	0.00	0.14
	sports facility density	The area of community sports facilities per unit area of HJD (total area of sports facilities in hangjungdong (km^2^)/total area of hangjungdong (km^2^))	Digital map		0.90	1.68	0.00	7.14
	school playground density	The area of school playgrounds per unit area of HJD (total area of school playgrounds in hangjungdong (km^2^)/total area of hangjungdong (km^2^))	Digital map		3.05	1.88	0.00	8.89
**Socioeconomic status**	household income	average monthly household income (Korean currency won)	KNHNES	577	488.32	338.94	17.00	1500.00
	education	total years of education completed	KNHNES	577	3.24	0.93	1.00	4.00
**Dietary pattern**	eat out	Average number of days on which resident eats out (days/week)	KNHNES	577	3.67	1.69	1.00	7.00
	energy	Amount of daily energy intake (amount of calories(kcal)/day)	KNHNES	577	1856.32	655.89	489.00	4152.00
	carbohydrate	Amount of daily carbohydrate intake (amount of carbohydrate (g)/day)	KNHNES	577	291.05	97.56	65.00	653.00
	fat	Amount of daily fat intake (amount of fat (g)/day)	KNHNES	577	38.62	19.88	6.00	111.00
	sodium	Amount of daily sodium intake (amount of sodium (mg)/day)	KNHNES	577	3051.52	1377.62	525.00	7985.00
**Physical activity**	weekly vigorous work	Weekly vigorous occupational physical activities (PA) (average number of days of vigorous occupational PA ^1^/week)	KNHNES	577	0.03	0.339	0	5
	weekly moderate work	Weekly moderate occupational PA (average number of days of moderate occupational PA ^2^/week)	KNHNES	577	0.44	1.402	0	7
	daily vigorous work	Daily vigorous occupational PA (average hours of vigorous occupational PA/day)	KNHNES	577	0.013	0.153	0	3
	daily moderate work	Daily moderate occupational PA (average hours of moderate occupational PA/day)	KNHNES	577	0.123	0.510	0	7
	weekly vigorous recreation	Weekly vigorous recreational PA per day (average number of days of vigorous recreational PA ^3^/week)	KNHNES	577	−0.06	1.29	−0.58	5.42
	weekly moderate recreation	Weekly moderate recreational PA per day (average number of days of moderate recreational PA ^4^/week)	KNHNES	577	−0.04	1.75	−1.09	5.91
	daily vigorous recreation	Daily vigorous recreational PA per day (average hours of vigorous recreational PA/day)	KNHNES	577	0.138	0.371	0	3
	daily moderate recreation	Daily moderate recreational PA per day (average hours of moderate recreational PA/day)	KNHNES	577	0.273	0.512	0	5
	weekly active commuting	Weekly active commuting (average number of days of active commuting ^5^/week)	KNHNES	577	3.43	2.68	0.00	7.00
	daily active commuting	Daily active commuting per day (average number of days of active commuting/day)	KNHNES	577	0.49	0.51	0.00	4.00
	weekly walking	Weekly walking per day (average number of days of walking exercise/week)	KNHNES	577	5.51	2.42	1.00	8.00

^1^ Vigorous occupational PA: heavy lifting over 20 kg, construction work, carrying heavy items by stairs etc.; ^2^ Moderate occupational PA: fast walking, carrying light items, cleaning, caring a baby etc.; ^3^ Vigorous recreational PA: running, jumping rope, basketball game, swimming, badminton, etc.; ^4^ Moderate recreational PA: walking, light jogging, weight training, golf, dance, Pilates, etc.; ^5^ Active commuting: commuting by walking and public transportation.
