# Erratum: Do the Walkability and Urban Leisure Amenities of Neighborhoods Affect the Body Mass Index of Individuals? Based on a Case Study in Seoul, South Korea

**DOI:** 10.3390/ijerph17145187

**Published:** 2020-07-17

**Authors:** Yunwon Choi, Heeyeun Yoon

**Affiliations:** 1 Interdisciplinary Program in Landscape Architecture, Seoul National University, 1 Gwanak-ro, Gwanak-gu, Seoul 151-742, Korea; yunwon.choi@snu.ac.kr; 2 Department of Landscape Architecture and Rural Systems Engineering, College of Agriculture and Life Sciences, Seoul National University, 1 Gwanak-ro, Gwanak-gu, Seoul 151-921, Korea; 3 Research Institute of Agriculture and Life Sciences, Seoul National University, 1 Gwanak-ro, Gwanak-gu, Seoul 151-921, Korea

The grant number in the acknowledgement was incorrect. The correction is provided below.

## 1. Incorrect Grant Number

This work was supported by the BK 21 Plus Project (Seoul National University Interdisciplinary Program in Landscape Architecture, Global leadership program toward innovative green infrastructure); the Korea Environmental Industry and Technology Institute (KEITI) (Grant number: 2014-001-310007); the Ministry of Education of the Republic of Korea and the National Research Foundation of Korea (Grant number: NRF-2017S1A5A8020226); the Creative-Pioneering Researchers Program through Seoul National University (Grant number: 500-20170162); the Ministry of Education of the Republic of Korea and the National Research Foundation of Korea (Grant number: NRF-2017S1A3A2066771); and the Korea Environmental Industry and Technology Institute (KEITI) through the Climate Change Response Technology Project, funded by the Korea Ministry of Environment (MOE) (201800131002).

## 2. Correct Grant Number

This work was supported by the Creative-Pioneering Researchers Program through Seoul National University (Grant number: 500-20170162), the Ministry of Education of the Republic of Korea and the National Research Foundation of Korea (Grant number: NRF-2017S1A3A2066771) and the Korea Environmental Industry and Technology Institute (KEITI) (Grant number: 2014-001-310007).

